# A point mutation in a *wspF-*like gene in *Pseudoalteromonas lipolytica* enhances the anticorrosion activity

**DOI:** 10.1128/aem.02154-24

**Published:** 2025-01-28

**Authors:** Zhenshun Zeng, Dan He, Zhiying Zhao, Tianci He, Qian Li, Yuqi Wang

**Affiliations:** 1 Key Laboratory for Water Quality and Conservation of the Pearl River Delta, Ministry of Education, School of Environmental Science and Engineering, Guangzhou University 605393 , Guangzhou, China; 2 State Key Laboratory of Applied Microbiology Southern China, Guangdong Provincial Key Laboratory of Microbial Culture Collection and Application, Guangdong Institute of Microbiology, Guangdong Academy of Sciences 639038 , Guangzhou, China; Washington University in St. Louis, St. Louis, Missouri, USA

**Keywords:** *Pseudoalteromonas*, genetic variant, biomineralization, anticorrosion

## Abstract

The protection of steel based on microbial biomineralization has emerged as a novel and eco-friendly strategy for corrosion control. However, the molecular basis of the biomineralization process in mineralization bacteria remains largely unexplored. We previously reported that *Pseudoalteromonas lipolytica* EPS+ strain provides protection against steel corrosion by forming a hybrid biomineralization film. In this study, we identified that a point mutation in the *AT00_08765* (*wspF-*like) gene, responsible for encoding a chemotaxis protein that regulates swimming motility and polysaccharide production, is linked to the observed anticorrosion activity in EPS+ strain. The engineered point mutation mutant strain, designated Δ*08765(707A*), exhibited similar phenotypes to the EPS+ strain, including colony morphology and cellulose production. Importantly, we demonstrated that moderate swimming motility in Δ*08765(707A*) plays a pivotal role in the development of a protective mineralization film on the steel surface. Additionally, we found that Δ*08765(707A*) enhances biofilm formation by rapidly forming small aggregates in the initial stage of biofilm growth. This process facilitated the assembly of more compact and larger mineralization products, effectively inhibiting steel corrosion. In addition, Δ*08765(707A*) formed a uniform mineralization film that completely covered the steel surface, preventing the formation of sheet-like steel corrosion products. Therefore, this study demonstrates that an engineering strain carrying a point mutation in the *AT00_08765* gene can significantly enhance the anticorrosion activity. This enhancement is accomplished through the formation of small bacteria-induced aggregates, followed by the development of larger mineralization products and the creation of a uniform organic–inorganic hybrid film.

**IMPORTANCE:**

In this study, we revealed that moderate swimming motility significantly influences the anticorrosion activity of marine *Pseudoalteromonas*. Furthermore, our study demonstrated that overproduction of cellulose facilitates cell aggregation rapidly during the initial stages of biofilm formation, thereby promoting the development of larger mineralization products and the formation of a uniform organic–inorganic hybrid film on the steel surface. Our findings provide new insights into the biomineralization mechanisms in *Pseudoalteromonas lipolytica*, potentially catalyzing the advancement of an eco-friendly microbial-driven approach to marine corrosion prevention.

## INTRODUCTION

Steel corrosion in marine environments is a critical issue, arising from the intricate interaction of chemical reactions, electrical process, and microbial activity (1). This complex set of factors can lead to substantial economic losses and pose safety hazards. While assessing the total cost of corrosion is a challenging task, a conservative estimate suggests that approximately 2.5 trillion US dollars are spent annually on direct corrosion expenses (2). This highlights the urgent need for effective strategies to mitigate corrosion rates and safeguard metal structures in marine environments.

In marine settings, submerged steel surfaces are quickly colonized by a plethora of microorganisms, with microbially influenced corrosion (MIC) being a significant cause of catastrophic corrosion failures (3, 4). Microorganisms, such as sulfate-reducing bacteria (SRB) and nitrate-reducing bacteria (NRB), colonize steel surface, which can lead to the formation of biofilm (5, 6). These biofilms have the potential to initiate or accelerate corrosion reactions by engaging in the electron transfer processes of the metal, consequently modifying the electrochemical properties of the steel surfaces (7). Several theories have been proposed to explain MIC corrosion mechanisms, including biocatalytic cathodic sulfate reduction (BCSR), metabolite-influenced corrosion (MMIC), and electrical microbial-influenced corrosion (EMIC) (8, 9). Additionally, metal-depositing bacteria, such as iron-oxidizing bacteria (FeOB) and manganese-oxidizing bacteria (MnOB), colonize steel surface, which can induce patchy biofilm formation, leading to deposit corrosion by creating an oxygen concentration difference cell (10, 11).

While biofilm formation by corrosion-causing bacteria can initiate or accelerate corrosion, some microorganisms, such as *Vibrio* sp. (12, 13), *Tenacibaculum* sp. (14, 15), and *Escherichia coli* (16) can induce corrosion inhibition. These organisms primarily achieve this through the neutralization of corrosive substances, the formation of protective films, or the stabilization of existing protective films on steel surfaces (17). Moreover, a novel and environmentally benign strategy has recently emerged, leveraging microbially induced calcium carbonate precipitation (MICP) to reduce steel corrosion (18–20). This strategy harnesses the MICP process to facilitate the transformation of an unstable bacterial biofilm into a homogeneous and stable biomineralized film on steel surfaces. Acting as an eco-friendly diffusion barrier, this biomineralized layer offers a protective shield against oxygen, chloride ions, and bacteria that contribute to corrosion, thereby exhibiting a substantial inhibitory effect on the corrosion process (21–23).

*Pseudoalteromonas*, prevalent in marine environments, exhibits a capacity to colonize diverse marine surfaces through the production of abundant extracellular polymeric substances (EPSs) (24, 25). Recent studies have shown that a genetic variant of *Pseudoalteromonas lipolytica* with cellulose overproduction, denoted as EPS+, exhibits robust anticorrosion activity through the bioprecipitation of carbonates on steel surface (26, 27). Despite the demonstrated efficacy of EPS+ in anticorrosion applications, the precise molecular determinants conferring protection against steel corrosion remain unclear.

The EPS+ variant underwent comprehensive whole-genome sequencing, revealing only three non-synonymous or non-sense mutations when aligned with the genome of the wild-type *P. lipolytica* (28). Among these mutations, a non-sense mutation was identified within the *AT00_08765* gene, which encodes a putative methylesterase sharing 30% similarity (98% coverage) with the WspF protein of *Pseudomonas aeruginosa* (29). Notably, in-frame deletion of *AT00_0876*5 (*wspF-*like) causes a change from smooth to wrinkled morphology due to the induction of cellulose production, mirroring the observed phenotypes of the EPS+ variant (30). Herein, we further examine and discuss the effect of the *wspF-*like gene on anticorrosion activity based on the formation of the protective biomineralization film on steel surface.

## RESULTS

### *P. lipolytica* Δ*08765* shares similar phenotypes with EPS+ strain except for anticorrosion activity

**Fig 1:**
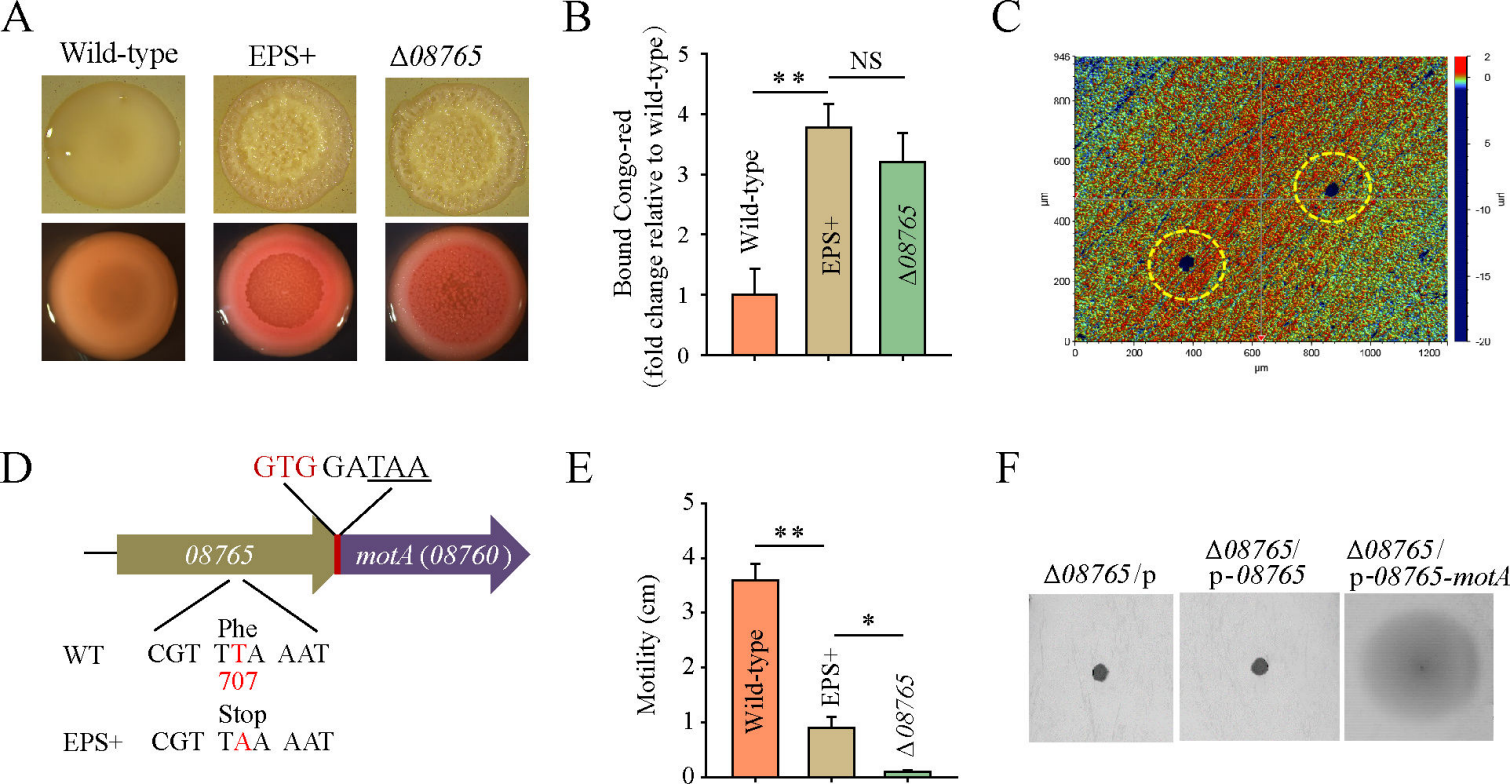
*P. lipolytica* Δ*08765* shares similar phenotypes with EPS+ strain except for swimming activity. (**A**) Colony morphologies were shown for different strains on seawater Luria Bertani (SWLB) agar medium with or without Congo red. (**B**) Cellulose productions were assessed for different strains using the Congo red binding assay. (**C**) Pitting corrosion on the steel surface was examined for the Δ*08765* strain by optical profilometry after performing an immersion test on day 14. The corrosion pits are indicated in yellow circles. (**D**) The coding sequences of *AT00_08760* and *motA* were found to overlap in the genome of *P. lipolytica*. (**E**) Swimming motility was examined for different strains by measuring the swimming zone achieved after 24 hours of incubation on 2216E containing 0.25% agar. (**F**) Swimming motility was examined for the Δ*08765* strain expressing either an empty vector pBBR1MCS (P) or complementation vectors. Data were obtained from three independent cultures, and the error bar represents the standard deviations (^∗^, *P* < 0.05; ^∗∗^, *P* < 0.01; NS, not significant). Images shown in Figure 1 to 7 are representative images.

We previously demonstrated that a biofilm-derived variant strain of *P. lipolytica*, designated EPS+, confers a strong steel anticorrosion activity, primarily attributed to the elevated production of cellulose (26). EPS+ carries a point mutation in *AT00_08765* (*wspF-*like), a gene associated with the chemotaxis signaling pathway controlling swimming motility and polysaccharide production (28). Deletion of the *AT00_08765* gene in wild-type *P. lipolytica* strain results in similar phenotypes to the EPS+ strain, characterized by wrinkled colony morphology and enhanced cellulose production (Fig. 1A and B). However, we observed that the *AT00_08765* mutant strain showed a reduced ability to inhibit pitting corrosion on the steel surface. The average sizes of the pits were approximately 38 µm in diameter and 20 µm in depth (Fig. 1C).

To explore the molecular basis underlying the anticorrosion activity of the EPS+ strain, we scrutinized the specific mutation locus in the genome of the EPS+. As shown in Fig. 1D, the EPS+ strain’s genome substitutes the base T with A at site 707 in the open reading frame (ORF) of *AT00_08765*, resulting in a stop codon at 236th amino acid and subsequent abolition of AT00_08765 expression. In contrast, the engineering *AT00_08765* deletion mutant deleted the whole ORF of the *AT00_08765*, inadvertently encompassing the start codon of the downstream *AT00_08760* (*motA*) due to sequence overlap (GTGGATAA) between the two adjacent genes (Fig. 1D). The *motA* gene encodes proton-powered stator units that are required for flagellar motility (31). Therefore, the deletion of *AT00_08765* might exert a polar effect on the expression of MotA, leading to a deficiency in motility. As anticipated, the Δ*08765* strain completely lost swimming motility, while the EPS+ strain retained swimming motility (Fig. 1E). To confirm that the Δ*08765* strain imposed a negative effect on the transcription of the downstream gene *motA*, two expression plasmids pBBR1MCS-*08765* and pBBR1MCS-*08765-motA* (co-expression of *AT00_08765* and *motA*) were constructed and transferred into the Δ*08765* strain, respectively. Results demonstrated that complementation with *AT00_08765-motA* fully restored swimming motility in the Δ*08765* strain, whereas complementation with *AT00_08765* alone had no effect on the recovery of swimming activity (Fig. 1F). Taken together, we have shown that the Δ*08765* strain exhibits similar phenotypes to the EPS+ strain in terms of colony morphology and cellulose production. However, the Δ*08765* strain completely loses swimming activity while the EPS+ retains a moderate swimming activity, suggesting that this discrepancy may be a key factor linked to the different performance on steel anticorrosion activity.

### A point mutation in *wspF-*like gene retains a moderate swimming motility

**Fig 2:**
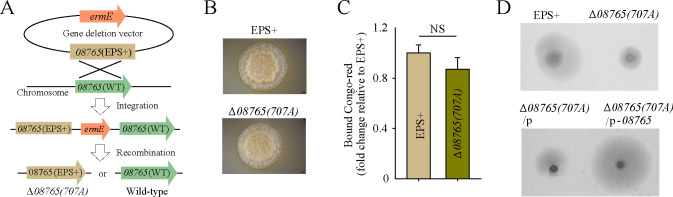
A point mutation in *AT00_08765* retains a moderate swimming motility. (**A**) The Δ*08765(707A*) mutant strain with a point mutation at site 707 (T to A) in the ORF of *AT00_08765* was constructed through gene homologous recombination. (**B**) Colony morphologies were shown for EPS+ and Δ*08765(707A*) strains on SWLB medium. (**C**) Cellulose productions were assessed for EPS+ and Δ*08765(707A*) strains using the Congo red binding assay. (**D**) Swimming motility was examined for EPS+ and Δ*08765(707A*) strains, as well as for Δ*08765(707A*) strain expressing an empty vector pBBR1MCS-cm and Δ*08765(707A*) strain expressing a complementation vector pBBR1MCS-Cm-*08765*.

As the Δ*08765* strain disrupts the expression of MotA, crucial for motility, we hypothesize that a single gene mutation in the *wspF-*like gene, without disrupting *motA* gene, may significantly contribute to steel anticorrosion. To generate a strain with a single gene mutation in *wspF-*like gene, we firstly constructed a gene deletion vector containing the variant *AT00_08765* (EPS+) gene fragment, amplified from the EPS+ strain. The resulting recombinant deletion vector was then introduced into the wild-type strain to facilitate the construction of the engineering strain (Fig. 2A). The constructed strain was designated Δ*08765(707A*), featuring a point mutation at site 707 (T to A) in the ORF of *AT00_08765*. As anticipated, the Δ*08765(707A*) strain exhibited a wrinkled colony morphology similar to that of the EPS+ strain (Fig. 2B). Moreover, the Δ*08765(707A*) strain produced comparable amounts of cellulose, mirroring the production observed in the EPS+ strain (Fig. 2C). Importantly, we observed that Δ*08765(707A*) retains a moderate swimming motility (Fig. 2D), suggesting the normal expression of the adjacent downstream gene, *motA*. To further confirm that the point mutation in *AT00_08765* affects swimming motility, the expression plasmid pBBR1MCS-*08765* was introduced into the Δ*08765(707A*). Results demonstrated that complementation with *AT00_08765* fully restored the swimming motility, whereas complementation with the empty vector had no effect on swimming motility in the Δ*08765(707A*) strain (Fig. 2D). Taken together, these results illustrate that the engineered point mutation strain, Δ*08765(707A*), shares similar phenotypes in terms of colony morphology and cellulose production with the EPS+ strain while retaining a moderate swimming motility.

### Δ*08765(707A)* enhances biofilm formation

**Fig 3:**
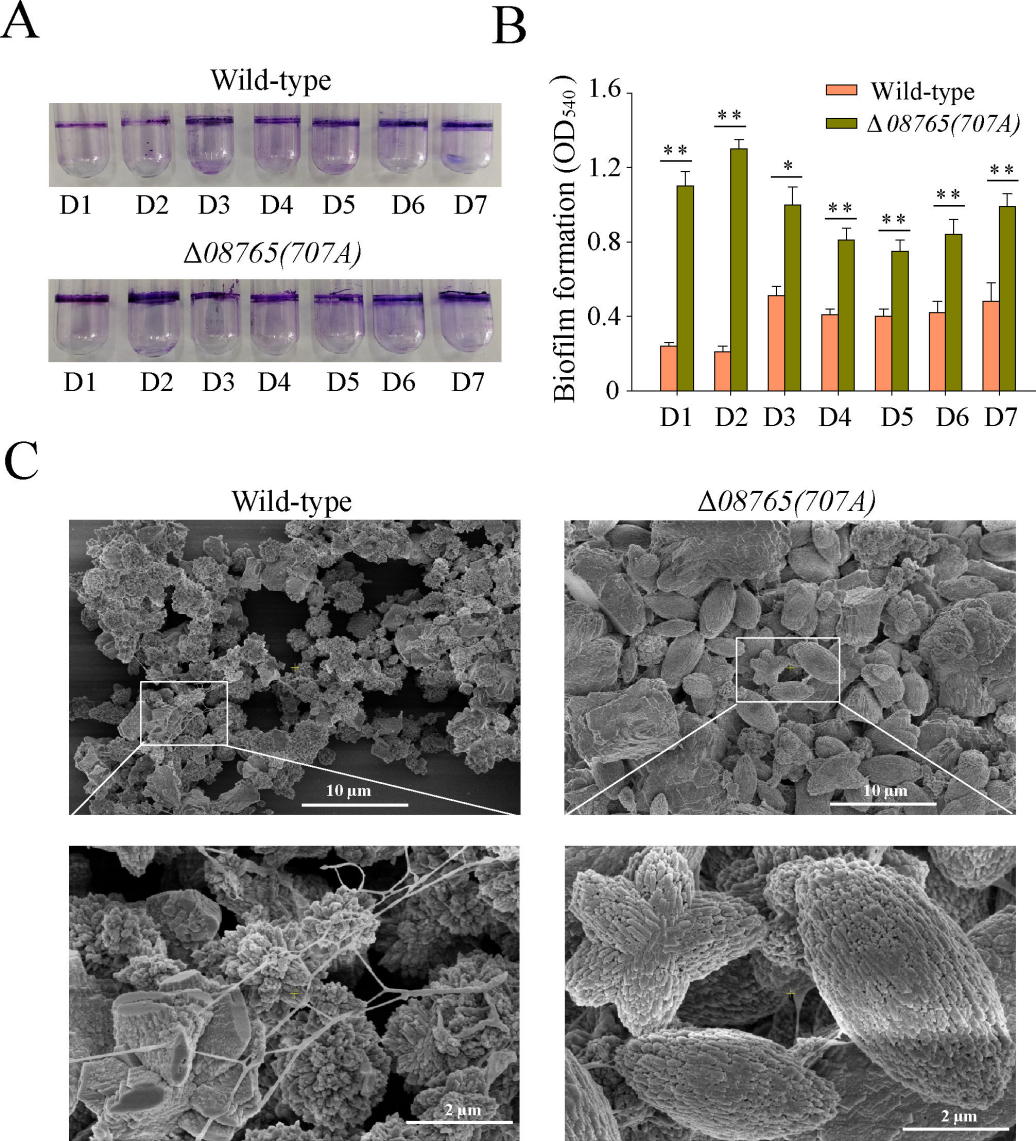
Δ*08765(707A*) enhances attachment biofilm formation. (**A**) Attachment biofilms were examined by crystal violet staining for wild-type and Δ*08765(707A*) strains from day 1 to day 7. (**B**) Quantification of attachment biofilms for wild-type and Δ*08765(707A*) strains from day 1 to day 7. (**C**) Attached products on glass surface were examined by SEM with the magnification of ×3,000 and ×15,000 for wild-type and Δ*08765(707A*) strains cultured for 7 days in a shaking incubator at 120 rpm.

Biofilm formation is a prerequisite for developing an organic–inorganic hybrid film that provides protection against steel corrosion (14, 16). Thus, we postulate that the Δ*08765(707A*) strain may also manifest a heightened biofilm. To assess biofilm formation, we initially conducted the crystal violet staining assay to evaluate the attached biofilm. The results revealed a significant increase in the attached biofilm for the Δ*08765(707A*) strain, approximately 4.6 ± 0.2-fold and 2.1 ± 0.2-fold higher than that of the wild-type strain after static incubation for day 1 and day 7 (Fig. 3A and B). In addition, the attached biofilm on day 7 also underwent examination via scanning electron microscopy (SEM). As shown in Fig. 3C, the wild-type strain formed a loosely aggregated structure with an irregular shape. In contrast, the Δ*08765(707A*) strain exhibited a dense and compact aggregate with a crystal-like structure. Elemental mapping showed that the aggregates of the wild-type and Δ*08765(707A*) strains comprised approximately 24.72% and 34.02% calcium (wt%), respectively, indicating the effective generation of mineralization products after 7 days of incubation (Fig. S1).

**Fig 4:**
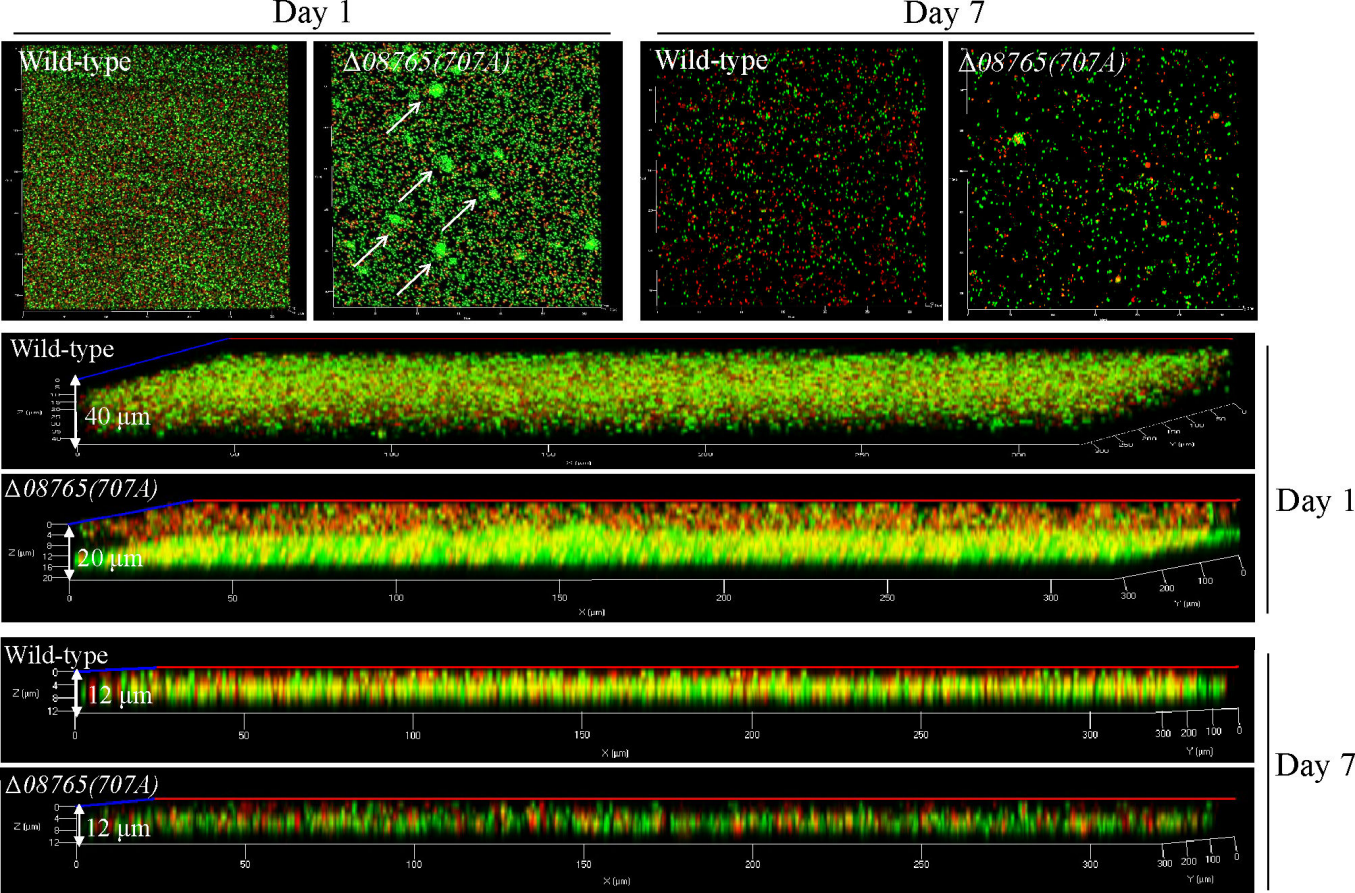
Δ*08765(707A*) promotes the formation of small aggregates. Biofilms stained with the LIVE/DEAD viability stain were examined on glass surface by CLSM after static incubation with wild-type or Δ*08765(707A*) strains for different days. A green color indicates viable (live) cells, and a red color indicates nonviable (dead) cells.

Next, the development of the attached biofilm was further examined using confocal laser scanning microscopy (CLSM) with live/dead staining. As shown in Fig. 4, the wild-type strain grew a dense and uniform biofilm with an approximate thickness of 40 µm on day 1, whereas the Δ*08765(707A*) strain developed a less dense biofilm with a thickness of nearly 20 µm but also a substantial proportion of red cells. However, on day 1, the Δ*08765(707A*) strain exhibited the formation of a small number of aggregates that appeared dark green, suggesting a robust bacterial activity conducive to the development of a substantial crystal-like structure. Upon the prolonged incubation without nutrient replenishment, both the biofilms of the wild-type strain and Δ*08765(707A*) strain experienced noticeable degradation by day 7 (Fig. 4). Taken together, these findings indicate that Δ*08765(707A*) has the capability to form a dense and compact biofilm by rapidly generating small aggregates during the initial biofilm growth stage, a phenomenon crucial for initiating the late stage of biofilm-induced calcification.

### Δ*08765(707A)* reduces pitting corrosion on the steel surface

**Fig 5:**
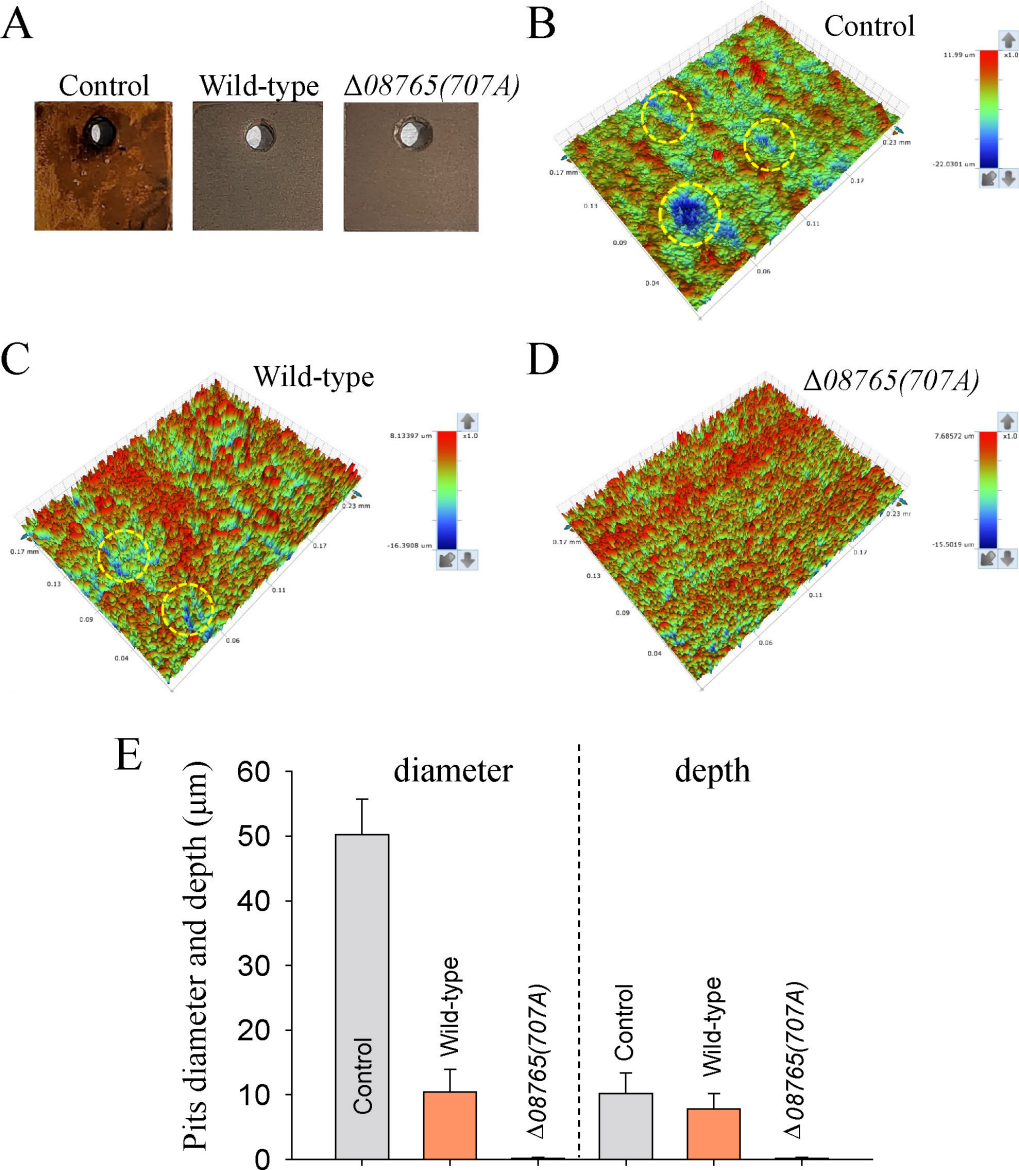
Δ*08765(707A*) reduces pitting corrosion on the steel surface. (**A**) Images of the carbon steel after immersion testing on day 7. Pitting corrosion on the steel surface was examined by optical profilometry after immersion testing for the control group (**B**), wild-type group (**C**), and Δ*08765(707A*) group (**D**). The color bar on the right side of the images indicates the depth variation of the corrosion pits. (E) Pit diameter and depth on the steel surface were measured after immersion testing.

To investigate the anticorrosion activities of the Δ*08765(707A*) strain, a carbon steel immersion test was conducted by inoculating different *P. lipolytica* strains into 40 mL of marine broth 2216E medium under a shaking condition. A control group, wherein no bacteria were introduced into the liquid medium, was also included. In the control group, the carbon steel surface exhibited extensive corrosion after 7 days of incubation. Conversely, for the carbon steels immersed in the culture of either wild-type *P. lipolytica* or Δ*08765(707A*) strains, both sets of the steel coupons appeared largely uncorroded upon visual inspection after 7 days of incubation period (Fig. 5A). To assess the efficiency of the steel protection, a 3D optical profile analysis was performed on the steel surface of different groups. The results revealed that the control group displayed numerous pits on the steel surface with approximately 50 µm in diameter and 10 µm in depth (Fig. 5B and E). In the wild-type *P. lipolytica* group, pitting corrosion was also observed on the steel surface, although the pits (approximately 10 µm in diameter and 8 µm in depth) appeared smaller than those in the control group (Fig. 5C and E). In contrast, the carbon steel inoculated with the Δ*08765(707A*) strain displayed an uncorroded surface without an evident pit corrosion (Fig. 5D). Taken together, these findings demonstrate that the Δ*08765(707A*) strain exhibits significant anticorrosion activity and can greatly reduce pitting corrosion on carbon steel compared to the wild-type strain.

### Δ*08765(707A)* forms more compact and larger mineralization products on the steel surface

**Fig 6:**
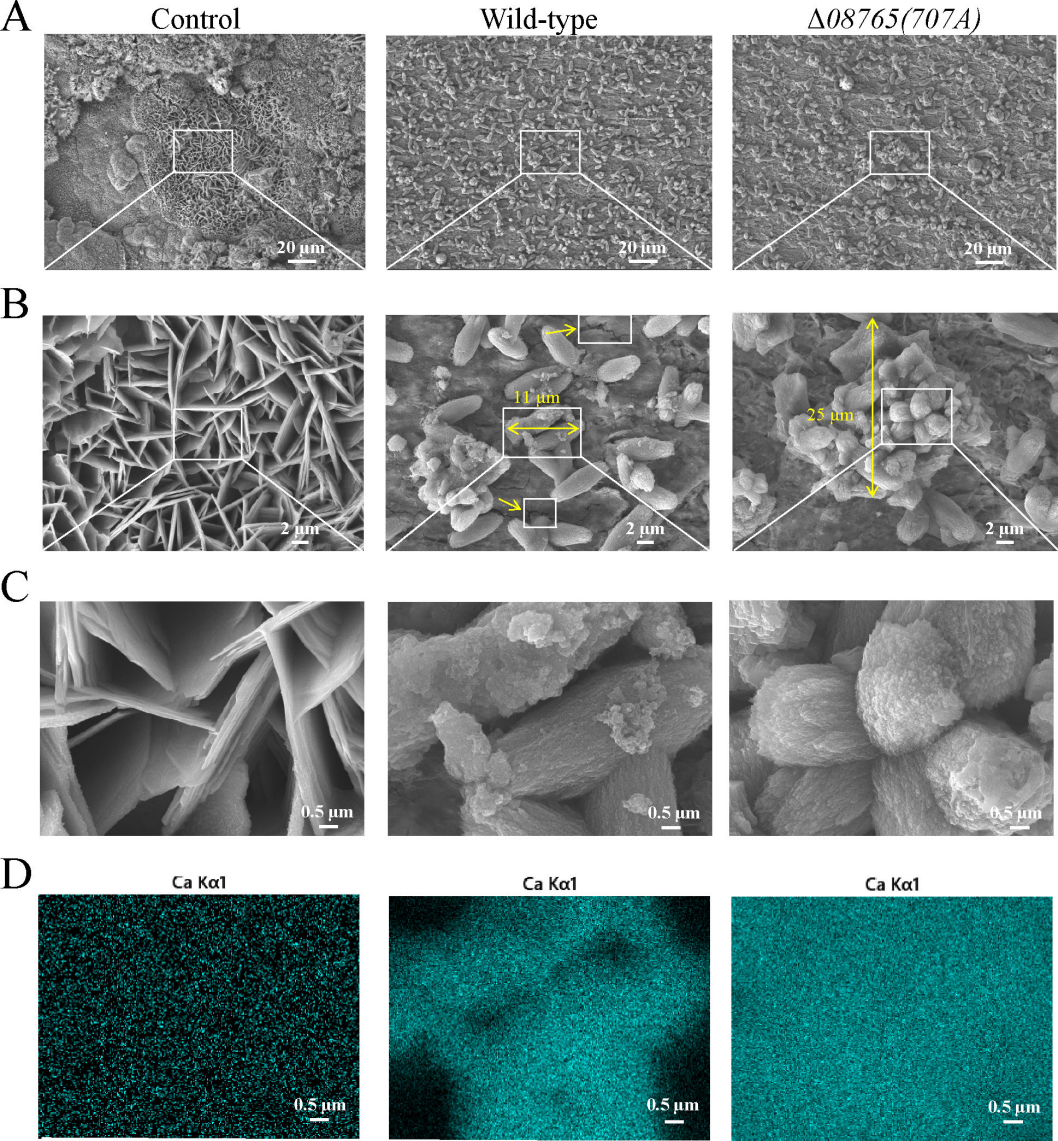
Δ*08765(707A*) forms more compact and larger mineralization products on the steel surface. Attached products on the steel surface were examined by SEM with the magnification of ×500 (**A**), ×3,000 (**B**), and ×15,000 (**C**) after immersion testing using wild-type and Δ*08765(707A*) strains. Single arrows indicate cracks where the biomineral layer does not completely cover the coupon on the film, while double arrows indicate the size of the mineralization products. (**D**) Elemental mappings of calcium are displayed in the lower panel of the image with maximum magnification. A brighter blue color is indicative of calcium.

*P. lipolytica* EPS+ strain provides protection against steel corrosion through the formation of organic–inorganic mineralized products (26). To examine the bacterial mineralization products on steel surfaces after 7 days of incubation, the morphologies of the steel surface were investigated for different groups using scanning electron microscopy. As shown in Fig. 6A, the control group, devoid of bacterial presence, exhibited a rough corrosion product on the steel surface, with the magnified view revealing a typical sheet-like corrosion product (Fig. 6B and C). In the wild-type group, a uniform bacteria-induced film was observed on the steel surface (Fig. 6A). However, upon magnification, small cracks (yellow arrow) were noticeable within the bacteria-induced film (Fig. 6B), suggesting the biomineral layer possesses an unstable surface structure that may lead to incomplete coverage of the steel coupon. Atop of the film, wild-type cells and their metabolites assembled to generate numerous large mineralized products (approximately 11 µm) containing approximately 25.4% (wt%) calcium, as confirmed by elemental mapping (Fig. 6D). In contrast to the wild-type group, carbon steel inoculated with the Δ*08765(707A*) strain displayed a biomineral layer without distinct cracks on the steel surface, which contains approximately 38.9% (wt%) calcium (Fig. 6D). Furthermore, the Δ*08765(707A*) strain exhibited a propensity to form larger mineralized aggregates (approximately 25 µm) on the steel surface compared to the wild-type strain (Fig. 6B). Taken together, *P. lipolytica* Δ*08765(707A*) demonstrates the ability to generate more compact and larger mineralization products on the steel surface that contributes to the improved anticorrosion activity.

### Δ*08765(707A)* forms a protective mineralization film on the steel surface

**Fig 7:**
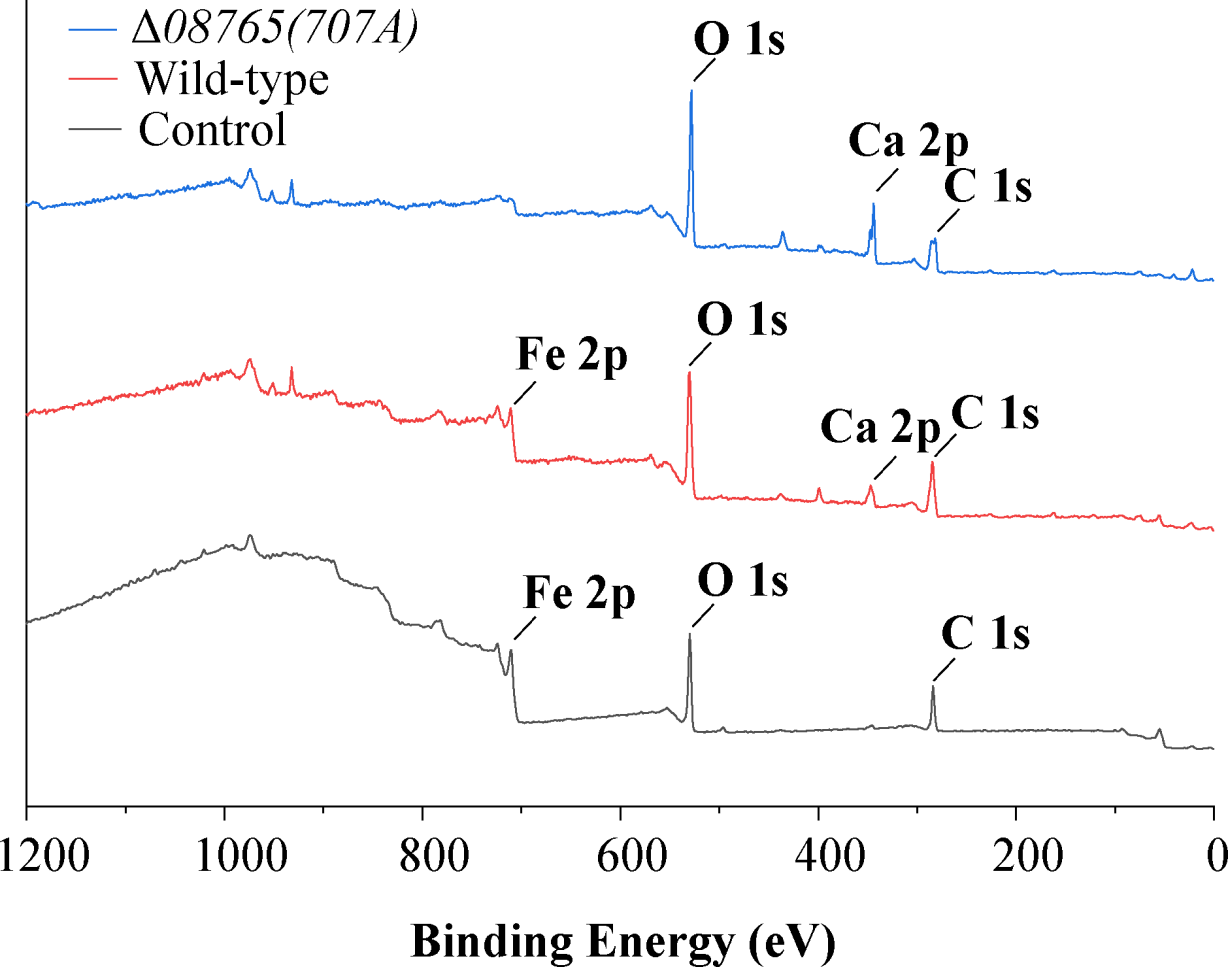
XPS examination of the mineralization film on steel surfaces. The properties of the steel surface were examined by XPS after immersion testing using wild-type and Δ*08765(707A*) strains.

To assess the extent of coverage by the mineralization film on the steel surface, X-ray photoelectron spectroscopy was employed to analyze the surface elements. As Fig. 7 shows, the control group without bacterial inoculation showed typical carbon and iron elements on the steel surface. When inoculated with the wild-type strain, a characteristic short calcium peak emerged due to the calcium carbonate mineralization. However, an iron element was also detected on the steel surface for the wild-type group, suggesting the incomplete coverage on the steel surface by the wild-type mineralization film. This observation aligns with the findings in Fig. 6, where small cracks were identified in the film formed by the wild-type strain. In contrast, the Δ*08765(707A*) group displayed a typical calcium peak similar to that of the wild-type group. Notably, the iron peak was scarcely detected on the steel surface, suggesting that the Δ*08765(707A*) strain is capable of forming a more efficient protective mineralization film compared to the wild-type strain.

**Fig 8:**
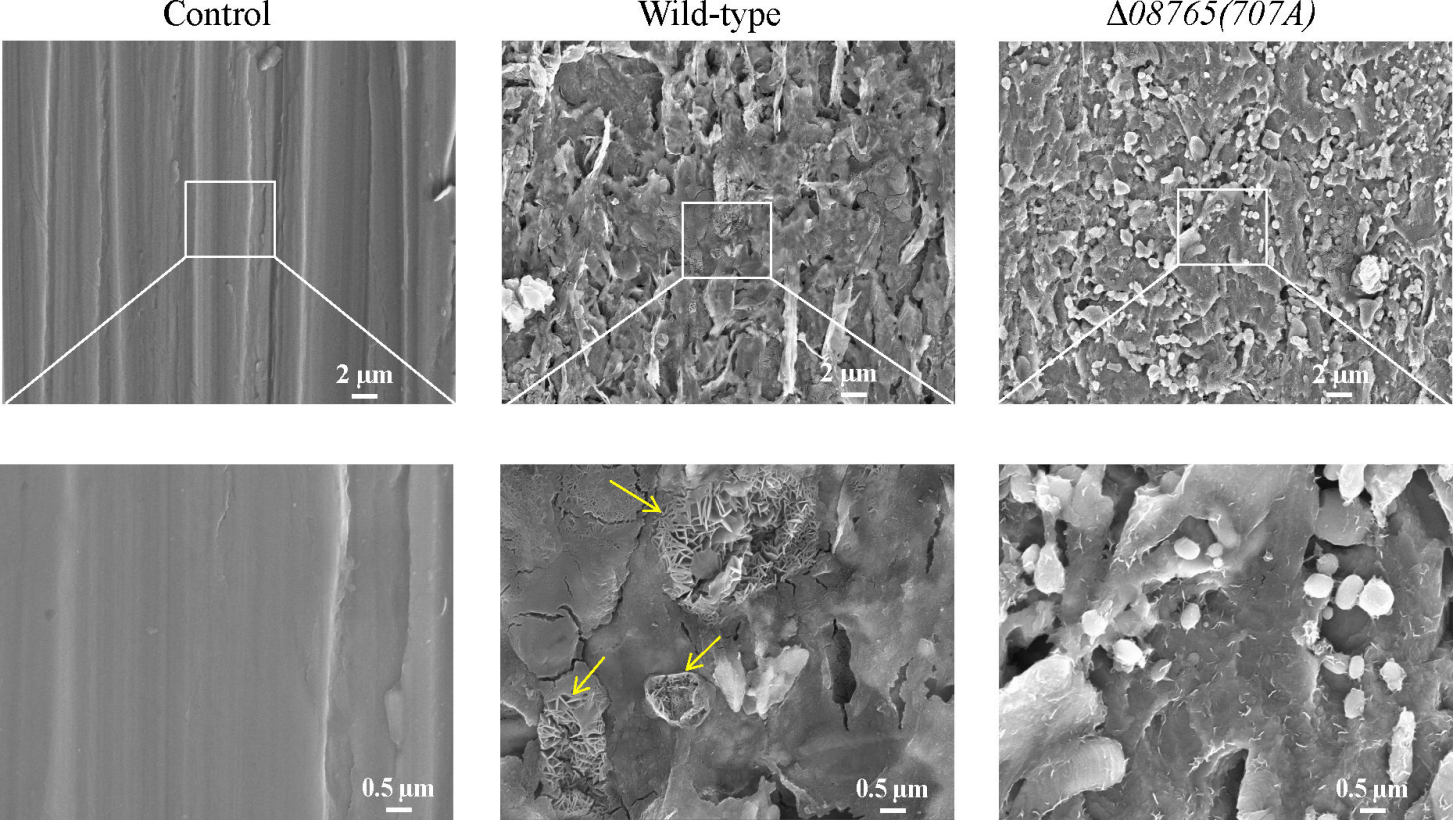
SEM examination on steel surfaces. Steel surfaces were examined by SEM with the magnification of ×3,000 (upper panel) and ×15,000 (lower panel) after removing the biomineralization products formed by wild-type and Δ*08765(707A*) strains.

To further investigate the anticorrosion effects of the wild-type and Δ*08765(707A*) groups, the mineralized products on carbon steel were removed by rinsing in a hydrochloric acid solution. Surface morphology examinations revealed that the control group, without bacterial inoculation, exhibited a smooth steel surface (Fig. 8, left panel). In contrast, the wild-type group still retained a bacteria-induced layer on the steel surface, while some corrosion products of the sheet-like iron oxide structure (yellow arrows) emerged after the removal of mineralized products (Fig. 8, middle panel). However, the steel surface in the Δ*08765(707A*) group remained covered by a protected layer without visible corrosion products (Fig. 8, right panel), indicating a significantly enhanced anticorrosion activity. Taken together, we have demonstrated that *P. lipolytica* Δ*08765(707A*) enhances the anticorrosion activity on steel surfaces by forming a uniform mineralization film on the steel surface.

## DISCUSSION

**Fig 9:**
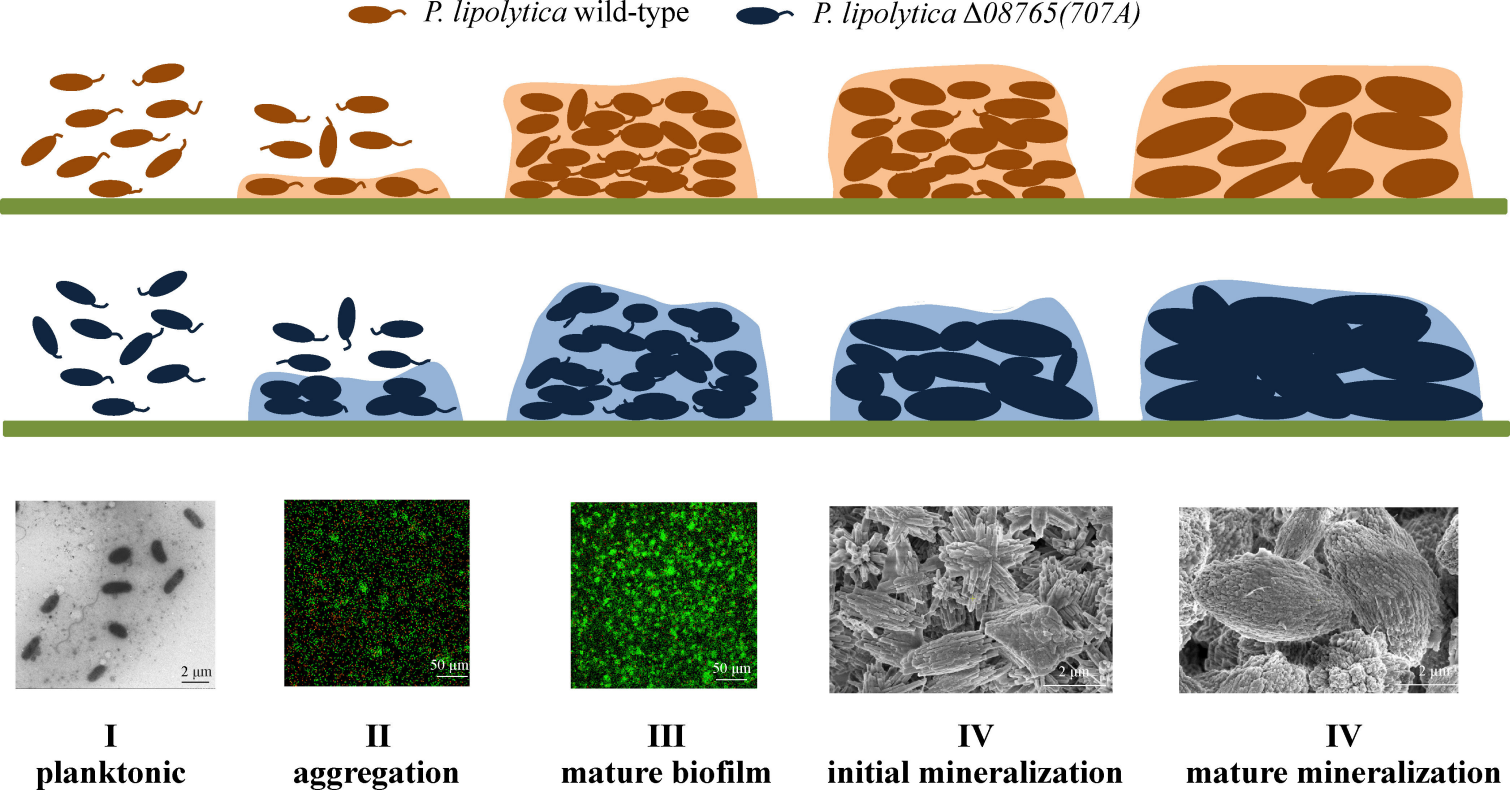
The proposed biomineralization process in Δ*08765(707A*). Δ*08765(707A*) cells exhibit a rapid aggregation during the initial stages of biofilm formation by overproducing cellulose. This aggregation subsequently promotes cell adhesion within the mature biofilm, which finally facilitates the development of larger mineralization products.

The gene *AT00_08765* encodes a putative methylesterase that exhibits a high sequence similarity to the WspF protein of *Pseudomonas aeruginosa*, a protein known to be involved in autoaggregation and the formation of wrinkled colony morphologies (32). In accordance with this, the inactivation of the *wspF-*like gene in *P. lipolytica* results in analogous autoaggregation and colony morphology (Fig. 1A; Fig. S2). Our study suggests that the engineering strain Δ*08765(707A*) enhances aggregation by overproducing cellulose during the initial phase of biofilm formation, which subsequently promotes adhesion within the mature biofilm. This increased cell adhesion facilitated the assembly of more compact and larger mineralization products, thereby effectively inhibiting corrosion on the steel surface (Fig. 9). Consequently, this study elucidates the molecular underpinnings of biomineralization in *P. lipolytica* and provides valuable insights into the development of genetically modified bacteria with enhanced anticorrosion properties.

Biofilm formation has been increasingly recognized as a protective strategy against steel corrosion (12, 14). During biofilm development, the initial colonization of suitable surfaces is facilitated by bacterial motility, a trait that is essential for this process. However, research indicates that these motile bacteria often become nonmotile upon transitioning to the biofilm state (33). This transition implies that motile bacteria must actively suppress their motility by reducing or inactivating flagella gene expression at the onset of biofilm formation to stabilize cell aggregates (34). Furthermore, genes associated with bacterial motility, such as flagellar regulatory genes *flrA*, *flrC*, and *flhA*, are highly susceptible to mutation during biofilm formation, leading to a reduction in motility activity (35). Although motility inhibition is crucial for biofilm formation, its relationship with steel anticorrosion is not well understood. A recent study indicates that flagella-associated genes are also downregulated during the process of biomineralization, suggesting that a decrease in motility might be a key factor in initiating biomineralization (36). In this study, we found that Δ*08765*, which lacks WspF-like and MotA, completely loses swimming motility and shows a reduced ability to inhibit pitting corrosion. However, Δ*08765(707A*), which lacks only WspF-like, retains moderate motility activity and exhibits an improved anticorrosion effect. These results suggest that bacteria need to retain motility activity to initiate surface colonization, while weak motility facilitates the formation of a protective biomineralization film, which benefits steel anticorrosion. To further corroborate this, two mutant strains, Δ*flhA* and Δ*fleQ*, which exhibit enhanced biofilm formation but are completely defective in swimming motility, were used in steel immersion tests. Results showed that both strains were ineffective in reducing pitting corrosion (Fig. S3). Therefore, swimming motility emerges as a key determinant in the formation of a protective biomineralization film in *P. lipolytica*.

The formation of a hybrid biomineralization film on the steel surface is a crucial process for effectively inhibiting corrosion (37). However, material properties, such as steel composition, significantly influence the formation of these protective film (38). For instance, *Pseudomonas stutzeri*, a common NRB strain, has been reported to accelerate the corrosion of the 1030 carbon steel and API 5LX carbon steel by inducing patchy biofilms on steel surface (39, 40). Conversely, *P. stutzeri* can form a protective film on low-carbon steel or AISI 304 stainless steel, which significantly reduces the corrosion rate (41, 42). Other studies also demonstrated that *P. stutzeri* can inhibit steel corrosion by forming a dense and compact iron mineralization film composed of nano Fe_3_O_4_ and FeOOH, or by forming a stable γ-Fe_2_O_3_ passivation films on X80 steel (43, 44). In addition to *P. stutzeri* and other NRBs, such as *Bacillus cereus*, have been reported to accelerate the pitting corrosion of X80 steel or 304 stainless steel through nitrate reduction (45, 46). However, other studies have reported that *B. cereus* can inhibit the corrosion of Q235 carbon steel or 316L stainless steel by forming a biofilm or biomineralization film that hinders extracellular electron transfer (47, 48). These studies revealed that microorganisms exhibit differences in biofilm or biomineralization formation on steels with varying composition. In our previous study, we demonstrated that carbon steel containing 0.2%~1.0% Mo element significantly induced the formation of mineralization products in *P. lipolytica* (49). Herein, we found that Δ*08765(707A*) can form a dense and more compact mineralization product layer on glass surfaces (Fig. 3B) but forms fewer mineralization products on Mo-free carbon steel surfaces (Fig. 5). In this study, to eliminate the influence of Mo element and facilitate a better phenotype comparison between wild-type *P. lipolytica* and its mutant strain, we conducted steel immersion tests using Mo-free carbon steel. Although Δ*08765(707A*) formed only a few larger mineralization products on Mo-free steel surfaces, the presence of the biomineralization film on the steel surface still offered protection from corrosion. In contrast, wild-type *P. lipolytica* formed fewer and smaller mineralization products on Mo-free steel surfaces, with distinguishable corrosion products when the biomineralization film was removed. Thus, our results demonstrate that a point mutation in *AT00_08765* in *P. lipolytica* significantly enhances anticorrosion activity on Mo-free steel surfaces.

MICP-based anticorrosion strategies are considered environmentally friendly and have garnered extensive attention in recent years. The characteristics of the mineralization products depend on the types of bacteria and their genome information, which confer specific metabolic activities (50). Several bacteria, including *P. lipolytica* (26), *Bacillus subtilis* (51), *Shewanella putrefaciens* (20), *Bacillus cereus* (47), *Paenibacillus* sp. (18), urease-producing bacteria (UPB) (21), and multi-species bacterial consortia (52), have the capability to form a protective biomineralization films with varying anticorrosion activity. To improve the stability and uniformity of the mineralization film formed by bacteria, the cross-application between corrosion science and molecular biology is imperative to further unravel the genetic basis of bacteria-induced mineralization.

## MATERIALS AND METHODS

### Strains and growth conditions

*Pseudoalteromonas lipolytica* SCSIO 04301 has been deposited in the Guangdong Microbiology Culture Center under the accession number GIMCC 1.82. All bacterial strains were stored at −80°C, and the frozen stocks were streaked onto fresh agar plates before conducting the experiments. *Escherichia coli* WM3064 was grown in LB medium supplemented with 0.3 mM DAP (2,6-diamino-pimelic acid) at 37°C. *Pseudoalteromonas lipolytica* strains were grown at 25°C in 2216E medium (Becton, Dickinson and Company, USA). The link to the composition of 2216E medium is https://www.dsmz.de/microorganisms/medium/pdf/DSMZ_Medium514.pdf. Kanamycin (50 mg/mL) and erythromycin (25 mg/mL) were used to maintain the gene knockout vector pK18mob*sacB*-ery in *E. coli* and *P. lipolytica*, respectively. Chloramphenicol (30 mg/mL) was used to maintain the expression vector pBBR1MCS-cm in both *E. coli* and *P. lipolytica*.

### Construction of the Δ*08765(707A)* strain

The Δ*08765(707A*) strain was constructed using our developed gene deletion method for marine *Pseudoalteromonas* strains with a minor modification (53). Briefly, the variant *AT00_08765* gene was PCR amplified from the genome of the EPS+ strain using the primer pair V08765-F/V08765-R. Subsequently, the amplified DNA fragment was ligated into the suicide plasmid pk18mob*sacB*-ery using the ClonExpress II One Step Cloning Kit (Vazyme, China) and then transferred into the *E. coli* WM3064. The recombinant plasmid was integrated into the chromosome of the wild-type *P. lipolytica* strain through conjugation. The Δ*08765(707A*) mutant strain was generated by screening for erythromycin resistance followed by *sacB*-based counterselection. The resulting mutant strain was further confirmed by sequencing the *AT00_08765* gene using the primer pair V08765-seqF/V08765-seqR. The prime sets used in this study are listed in Table S1.

### Gene complementation

The vector pBBR1MCS-Cm was used for gene expression in *P. lipolytica* (54). The *AT00_08765* and *AT00_08765-motA* genes with its native promoter were PCR amplified from the genome of the wild-type *P. lipolytica* using the primer pair p-*08765*-F/p-*08765*-R and p-*08765-motA*-F/p-*08765-motA*-R, respectively. The amplified DNA fragments were then ligated into the pBBR1MCS-Cm vector in the host of *E. coli* WM3064 and subsequently transferred to *P. lipolytica* mutant strain through conjugation. The resultant expression plasmids were further confirmed by sequencing using the primer sets pBBR1MCS-f/pBBR1MCS-r, which are listed in Table S1.

### Colony morphology observation

Cells were grown overnight in 2216E medium at 25°C. A 10 µL volume of the cultures was plated on sea water LB agar media and incubated for 3 days. Colony morphologies were imaged using a stereoscopic microscope.

### Congo red binding assay

A 10 µL volume of the fresh overnight cultures was spotted onto 2216E agar plates containing 100 µg/mL Congo red (CR) and was incubated at 25°C for 3 days. Increasing depth of color in the colony indicated high levels of cellulose matrix production. To quantify cellulose production, CR was added to 1 mL of the overnight culture at a final concentration of 50 µg/mL and was cultured with shaking condition for 3 hours. The cultures were then centrifuged at 15,000 rpm for 5 minutes. The color of the cell pellet was imaged, and the supernatant was collected. The amount of CR bound to the cells was determined by measuring the absorbance of the supernatant at 490 nm, as previously described (55).

### Carbon steel immersion test

Q235 carbon steel coupons with dimensions of 10 × 10 × 2 mm were used for all tests. Table S2 shows the chemical compositions (wt%) of Q235 carbon steel. Each coupon underwent sequential abrasion with 200, 400, 600, and 800 grit sandpapers, followed by rinsing with ethanol as previously described (26). Prior to each experiment, all coupons were dried under ultraviolet light for 30 minutes. Immersion testing was conducted in a 100 mL container, with a steel coupon submerged in 40 mL of 2216E. A volume of 0.4 mL of the fresh overnight cultures of each strain was inoculated into the container, which was then incubated in a shaking incubator at 120 rpm for 7 days or 14 days. Half of the medium was replaced with fresh 2216E medium every 2 days. A control group was also included without the addition of bacteria.

### Pitting corrosion examination

The steel coupons underwent an immersion test using different bacteria. Subsequently, corrosion products or the attached film on the steel coupons was removed by sequential washing in diluted HCl solution, saturated NaHCO_3_ solution, and deionized (DI) water, each step lasting half a minute. The blow-dried steel coupons were then examined using an optical profilometer (Bruker Contour GT-K, USA). The morphology of the pits was examined using optical profilometry at three different points, with a scanning area of 170 × 230 µm. The diameter and depth of the pits on the steel surface were analyzed using Vision64 software.

### Swimming motility

A 1 µL aliquot of the fresh overnight cultures was spotted onto 2216E plate containing 0.25% agar and incubated for 24 hours at room temperature. Subsequently, the plates were imaged, and the swimming zones where bacteria grew on the motility agar plates were measured by the diameter of the halos (56).

### Attached biofilm

Attached biofilm formation was assessed by growing the strains in glass tubes with crystal violet staining as reported previously (57). Briefly, cells were inoculated into 2 mL 2216E medium with an initial turbidity at 600 nm of 0.05 and were incubated for different periods of time without shaking. After incubation, the cultures were removed, and the attached biofilm was stained with crystal violet for 20 minutes. The attached biofilm was observed after the removal of crystal violet and rinsing three times with running water. To quantify the attachment biofilm, the crystal violet was dissolved by adding 3 mL of ethanol absolute for 30 minutes, and the dissolved solution was then measured at the absorption of 540 nm.

### Scanning electron microscopy visualization

The biomineralization or corrosion products formed on glass fragments (10 × 10 × 2 mm) or carbon steel were examined using SEM equipped with energy-dispersive X-ray spectroscopy (EDS) (TESCAN MIRA LMS, Czech Republic) as previously described (26). Briefly, a 30 µL volume of fresh overnight cultures was inoculated into a glass tube containing 3 mL of 2216E with a glass fragment. The glass fragments were collected after culturing with the bacteria in a shaking incubator at 120 rpm for 7 days, while the steel coupons underwent an immersion test. To assess the anticorrosion effect, the biomineralization film formed on carbon steel was removed by sequential washing in diluted HCl solution, saturated NaHCO_3_ solution, and DI water, each step lasting half a minute. All prepared samples were further processed with vacuum freeze-drying. The lyophilized samples were then supported on an aluminum stab covered with a conductive carbon tape and were directly observed after applying a coating of platinum.

### Confocal laser scanning microscopy visualization

A 10 µL volume of the fresh overnight cultures was inoculated into a 24-well plate containing a cell climbing slice with 1 mL of 2216E. The 24-well plate was incubated for different periods of time without shaking. Subsequently, the cell climbing slice was rinsed with 2216E and stained with the LIVE/DEAD *Bac*Light bacterial viability kits (Thermo Fisher Scientific, USA) in a dark environment. The sample was then examined using CLSM after fluorescent staining. Damaged membranes (dead) and intact membranes (live) were distinguished by displaying red and green colors, respectively.

### X-ray photoelectron spectroscopy examination

The steel coupons underwent processing through immersion testing. Additionally, a steel coupon without any treatment was included as a control group. Subsequently, the surface properties of the biomineralization film formed on carbon steel were examined using X-ray photoelectron spectroscopy (XPS). XPS analysis was conducted using the Thermo Scientific Escalab250. The spectrometer is equipped with an Al Kα X-ray source generating photons with an energy of 1,486.6 eV. All analyses were performed at the pass energy of 150 eV. The center area of each coupon surface was examined at a single point with a spot size of 400 µm.

### Statistical analysis

Statistical analyses were performed using GraphPad Prism v.8.0 software. Data are presented as mean ± standard deviation (SD) of three independent cultures. Statistical significance was assessed using a two-tailed unpaired Student’s *t*-test, and asterisks are used to represent statistically significant differences (58).

## Data Availability

The data that support the findings of this study are available from the corresponding author upon reasonable request.

## SUPPLEMENTAL MATERIAL

Supplemental material aem.02154-24-s0001.docx Tables S1 and S2; Figures S1 to S3.

ASM does not own the copyrights to Supplemental Material that may be linked to, or accessed through, an article. The authors have granted ASM a non-exclusive, world-wide license to publish the Supplemental Material files. Please contact the corresponding author directly for reuse.

## References

[1] Alcántara J, Fuente D de la, Chico B, Simancas J, Díaz I, Morcillo M. 2017. Marine atmospheric corrosion of carbon steel: a review. Materials (Basel) 10:406. doi:10.3390/ma10040406 28772766 PMC5506973

[2] Hou BR, Li XG, Ma XM, Du CW, Zhang DW, Zheng M, Xu WC, Lu DZ, Ma FB. 2017. The cost of corrosion in China. npj Mater Degrad 1:4. doi:10.1038/s41529-017-0005-2

[3] Liu L, Wu XD, Wang QH, Yan ZT, Wen X, Tang J, Li XM. 2022. An overview of microbiologically influenced corrosion: mechanisms and its control by microbes. Corros Rev 40:103–117. doi:10.1515/corrrev-2021-0039

[4] Little BJ, Blackwood DJ, Hinks J, Lauro FM, Marsili E, Okamoto A, Rice SA, Wade SA, Flemming HC. 2020. Microbially influenced corrosion—Any progress? Corros Sci 170:108641. doi:10.1016/j.corsci.2020.108641

[5] Y G A, Mulky L. 2023. Biofilms and beyond: a comprehensive review of the impact of Sulphate Reducing Bacteria on steel corrosion. Biofouling 39:897–915. doi:10.1080/08927014.2023.2284316 38073525

[6] Videla HA, Herrera LK. 2005. Microbiologically influenced corrosion: looking to the future. Int Microbiol 8:169–180. 16200495

[7] Wang D, Zhou EZ, Xu DK, Lovley DR. 2023. Burning question: are there sustainable strategies to prevent microbial metal corrosion? Microb Biotechnol 16:2026–2035. doi:10.1111/1751-7915.14347 37796110 PMC10616648

[8] Knisz J, Eckert R, Gieg LM, Koerdt A, Lee JS, Silva ER, Skovhus TL, An Stepec BA, Wade SA. 2023. Microbiologically influenced corrosion-more than just microorganisms. FEMS Microbiol Rev 47:1–33. doi:10.1093/femsre/fuad041 PMC10479746 37437902

[9] Liu P, Zhang HT, Fan YQ, Xu DK. 2023. Microbially influenced corrosion of steel in marine environments: a review from mechanisms to prevention. Microorganisms 11:2299. doi:10.3390/microorganisms11092299 37764143 PMC10535020

[10] Lee JS, Little BJ. 2019. A mechanistic approach to understanding microbiologically influenced corrosion by metal-depositing bacteria. CORR 75:6–11. doi:10.5006/2899

[11] Little BJ, Hinks J, Blackwood DJ. 2020. Microbially influenced corrosion: towards an interdisciplinary perspective on mechanisms. Int Biodeterior Biodegradation 154:105062. doi:10.1016/j.ibiod.2020.105062

[12] Gao Y, Feng DQ, Moradi M, Yang CT, Jin YT, Liu D, Xu DK, Chen XB, Wang FH. 2021. Inhibiting corrosion of aluminum alloy 5083 through Vibrio species biofilm. Corros Sci 180:109188. doi:10.1016/j.corsci.2020.109188

[13] Gao Y, Zhang MX, Fan YQ, Li Z, Cristiani P, Chen XB, Xu DK, Wang FH, Gu TY. 2022. Marine Vibrio spp. protect carbon steel against corrosion through secreting extracellular polymeric substances. npj Mater Degrad 6:6. doi:10.1038/s41529-021-00212-2

[14] Li Z, Xu Y, Zhang J, Feng D, Fan Y, Xu D, Wang F. 2023. Living marine bacterium Tenacibaculum mesophilum D-6 inhibits crevice corrosion of X70 carbon steel. Corros Sci 215:111012. doi:10.1016/j.corsci.2023.111012

[15] Li Z, Zhou JY, Yuan XY, Xu Y, Xu DK, Zhang DW, Feng DQ, Wang FH. 2021. Marine biofilms with significant corrosion inhibition performance by secreting extracellular polymeric substances. ACS Appl Mater Interfaces 13:47272–47282. doi:10.1021/acsami.1c14746 34570482

[16] Li Z, Ren Y, Li Z, Zhang J, Fan Y, Jiang G, Xu D, Gu T, Wang F. 2024. Engineered living biofilm with enhanced metal binding ability for corrosion protection in seawater. Adv Funct Mater 34:202313120. doi:10.1002/adfm.202313120

[17] Videla HA, Herrera LK. 2009. Understanding microbial inhibition of corrosion. A comprehensive overview. Int Biodeterior Biodegradation 63:896–900. doi:10.1016/j.ibiod.2009.02.002

[18] Fan WB, Qian CX, Rui YF. 2023. A strong inhibitory effect of microbe-induced mineralization on corrosion on steel surfaces. J of Mater Eng Perform 32:6957–6973. doi:10.1007/s11665-022-07586-7

[19] Shan XY, Wang J, Du M, Tian ZY. 2024. Inhibitory effect of marine Bacillus sp. and its biomineralization on the corrosion of X65 steel in offshore oilfield produced water. Bioelectrochemistry 157:108659. doi:10.1016/j.bioelechem.2024.108659 38330530

[20] Lou YT, Chang WW, Cui TY, Qian HC, Huang LY, Ma LW, Hao XP, Zhang DW. 2021. Microbiologically influenced corrosion inhibition of carbon steel via biomineralization induced by Shewanella putrefaciens. npj Mater Degrad 5:59. doi:10.1038/s41529-021-00206-0

[21] Sun XH, Wai OWH, Xie JW, Li XD. 2024. Biomineralization to prevent microbially induced corrosion on concrete for sustainable marine infrastructure. Environ Sci Technol 58:522–533. doi:10.1021/acs.est.3c04680 38052449 PMC10785763

[22] Ivanova LA, Egorov VV, Zabrodskaya YA, Shaldzhyan AA, Baranchikov Ay, Tsvigun NV, Lykholay AN, Yapryntsev AD, Lebedev DV, Kulminskaya AA. 2023. Matrix is everywhere: extracellular DNA is a link between biofilm and mineralization in Bacillus cereus planktonic lifestyle. NPJ Biofilms Microbiomes 9:9. doi:10.1038/s41522-023-00377-5 36854956 PMC9975174

[23] Lou YT, Chang WW, Huang LY, Chen XD, Hao XP, Qian HC, Zhang DW. 2024. Influence of marine Shewanella putrefaciens and mediated calcium deposition on Q235 carbon steel corrosion. Bioelectrochemistry 157:108657. doi:10.1016/j.bioelechem.2024.108657 38335713

[24] Holmström C, Kjelleberg S. 1999. Marine Pseudoalteromonas species are associated with higher organisms and produce biologically active extracellular agents. FEMS Microbiol Ecol 30:285–293. doi:10.1111/j.1574-6941.1999.tb00656.x 10568837

[25] Wu ZW, Wu YY, Huang YQ, He J, Su P, Feng DQ. 2021. Insights into the planktonic to sessile transition in a marine biofilm-forming Pseudoalteromonas isolate using comparative proteomic analysis. Aquat Microb Ecol 86:69–84. doi:10.3354/ame01959

[26] Liu T, Guo ZW, Zeng ZS, Guo N, Lei YH, Liu T, Sun SB, Chang XT, Yin YS, Wang XX. 2018. Marine bacteria provide lasting anticorrosion activity for steel via biofilm-induced mineralization. ACS Appl Mater Interfaces 10:40317–40327. doi:10.1021/acsami.8b14991 30335931

[27] Guo N, Zhao QY, Hui XR, Guo ZW, Dong YH, Yin YS, Zeng ZS, Liu T. 2022. Enhanced corrosion protection action of biofilms based on endogenous and exogenous bacterial cellulose. Corros Sci 194:109931. doi:10.1016/j.corsci.2021.109931

[28] Zeng ZS, Guo XP, Li BY, Wang PX, Cai XS, Tian XP, Zhang S, Yang JL, Wang XX. 2015. Characterization of self-generated variants in Pseudoalteromonas lipolytica biofilm with increased antifouling activities. Appl Microbiol Biotechnol 99:10127–10139. doi:10.1007/s00253-015-6865-x 26264135 PMC4643108

[29] He K, Bauer CE. 2014. Chemosensory signaling systems that control bacterial survival. Trends Microbiol 22:389–398. doi:10.1016/j.tim.2014.04.004 24794732 PMC4273944

[30] Zeng Z, Lin S, Li Q, Wang W, Wang Y, Xiao T, Guo Y. 2022. Molecular basis of wrinkled variants isolated from Pseudoalteromonas lipolytica biofilms. Front Microbiol 13:797197. doi:10.3389/fmicb.2022.797197 35295294 PMC8919034

[31] Wadhwa N, Berg HC. 2022. Bacterial motility: machinery and mechanisms. Nat Rev Microbiol 20:161–173. doi:10.1038/s41579-021-00626-4 34548639

[32] D’Argenio DA, Calfee MW, Rainey PB, Pesci EC. 2002. Autolysis and autoaggregation in Pseudomonas aeruginosa colony morphology mutants. J Bacteriol 184:6481–6489. doi:10.1128/JB.184.23.6481-6489.2002 12426335 PMC135425

[33] Houry A, Briandet R, Aymerich S, Gohar M. 2010. Involvement of motility and flagella in Bacillus cereus biofilm formation. Microbiol (Reading) 156:1009–1018. doi:10.1099/mic.0.034827-0 20035003

[34] Guttenplan SB, Kearns DB. 2013. Regulation of flagellar motility during biofilm formation. FEMS Microbiol Rev 37:849–871. doi:10.1111/1574-6976.12018 23480406 PMC3718880

[35] Luo M, Chen GZ, Yi CR, Xue BS, Yang XM, Ma Y, Qin ZX, Yan J, Liu XY, Liu Z. 2023. Dps-dependent in vivo mutation enhances long-term host adaptation in Vibrio cholerae. PLoS Pathog 19:e1011250. doi:10.1371/journal.ppat.1011250 36928244 PMC10104298

[36] Ma L, Pang AP, Luo YS, Lu XL, Lin FM. 2020. Beneficial factors for biomineralization by ureolytic bacterium. Microb Cell Fact 19:12. doi:10.1186/s12934-020-1281-z 31973723 PMC6979283

[37] Lou YT, Chang WW, Cui TY, Wang JK, Qian HC, Ma LW, Hao XP, Zhang DW. 2021. Microbiologically influenced corrosion inhibition mechanisms in corrosion protection: a review. Bioelectrochemistry 141:107883. doi:10.1016/j.bioelechem.2021.107883 34246844

[38] Liu Y, Ali A, Su JF, Li K, Hu RZ, Wang Z. 2023. Microbial-induced calcium carbonate precipitation: influencing factors, nucleation pathways, and application in waste water remediation. Sci Total Environ 860:160439. doi:10.1016/j.scitotenv.2022.160439 36574549

[39] Parthipan P, AlSalhi MS, Devanesan S, Rajasekar A. 2021. Evaluation of Syzygium aromaticum aqueous extract as an eco-friendly inhibitor for microbiologically influenced corrosion of carbon steel in oil reservoir environment. Bioprocess Biosyst Eng 44:1441–1452. doi:10.1007/s00449-021-02524-8 33710453

[40] Salgar-Chaparro SJ, Darwin A, Kaksonen AH, Machuca LL. 2020. Carbon steel corrosion by bacteria from failed seal rings at an offshore facility. Sci Rep 10:12287. doi:10.1038/s41598-020-69292-5 32703991 PMC7378185

[41] Gunasekaran G, Chongdar S, Gaonkar SN, Kumar P. 2004. Influence of bacteria on film formation inhibiting corrosion. Corros Sci 46:1953–1967. doi:10.1016/j.corsci.2003.10.023

[42] Recio-Hernandez J, Galicia-García M, Silva-Jiménez H, Malpica-Calderón R, Ordoñez- Casanova EG. 2021. EIS Evaluation of corrosion resistance of AISI 304 stainless steel exposed to Pseudomonas stutzeri. Int J Electrochem Sci 16:21058. doi:10.20964/2021.05.39

[43] Liu HX, Chen W, Tan Y, Meng GZ, Liu HF, Cheng Y, Liu HW. 2022. Characterizations of the biomineralization film caused by marine Pseudomonas stutzeri and its mechanistic effects on X80 pipeline steel corrosion. J Mater Sci Technol 125:15–28. doi:10.1016/j.jmst.2022.02.033

[44] Fu Q, Xu J, Wei BX, Qin QY, Bai YL, Yu CK, Sun C. 2022. Mechanistic diversity between nitrate and nitrite on biocorrosion of X80 pipeline steel caused by Desulfovibrio desulfurican and Pseudomonas stutzeri. Corros Sci 207:110573. doi:10.1016/j.corsci.2022.110573

[45] Wan HX, Song DD, Zhang DW, Du CW, Xu DK, Liu ZY, Ding D, Li XG. 2018. Corrosion effect of Bacillus cereus on X80 pipeline steel in a Beijing soil environment. Bioelectrochemistry 121:18–26. doi:10.1016/j.bioelechem.2017.12.011 29329018

[46] Yu S, Lou YT, Zhang DW, Zhou EZ, Li Z, Du CW, Qian HC, Xu DK, Gu TY. 2020. Microbiologically influenced corrosion of 304 stainless steel by nitrate reducing Bacillus cereus in simulated Beijing soil solution. Bioelectrochemistry 133:107477. doi:10.1016/j.bioelechem.2020.107477 32035394

[47] Hu YL, Chen CM, Liu ST, Zhou YR, Jia WB, Cao Y. 2023. Biofilm-induced corrosion inhibition of Q235 carbon steel by anaerobic Bacillus cereus inoculum in simulated cooling water. Environ Sci Pollut Res 30:20833–20848. doi:10.1007/s11356-022-23561-0 36260227

[48] Li SL, Li L, Qu Q, Kang YX, Zhu BL, Yu DT, Huang R. 2019. Extracellular electron transfer of Bacillus cereus biofilm and its effect on the corrosion behaviour of 316L stainless steel. Colloids Surf B Bio 173:139–147. doi:10.1016/j.colsurfb.2018.09.059 30278362

[49] Guo ZW, Wang WQ, Guo N, Zeng ZS, Liu T, Wang XX. 2019. Molybdenum-mediated chemotaxis of Pseudoalteromonas lipolytica enhances biofilm-induced mineralization on low alloy steel surface. Corros Sci 159:108123. doi:10.1016/j.corsci.2019.108123

[50] Lou YT, Chang WW, Cui TY, Qian HC, Hao XP, Zhang DW. 2023. Microbiologically influenced corrosion inhibition induced by S. putriefaciens mineralization under extracellular polymeric substance regulation via FlrA and FlhG genes. Corros Sci 221:111350. doi:10.1016/j.corsci.2023.111350

[51] Guo ZW, Pan S, Liu T, Zhao QY, Wang YN, Guo N, Chang XT, Liu T, Dong YH, Yin YS. 2019. Bacillus subtilis inhibits Vibrio natriegens-induced corrosion via biomineralization in seawater. Front Microbiol 10:1111. doi:10.3389/fmicb.2019.01111 31164881 PMC6536734

[52] Guo N, Wang YN, Hui XR, Zhao QY, Zeng ZS, Pan S, Guo ZW, Yin YS, Liu T. 2021. Marine bacteria inhibit corrosion of steel via synergistic biomineralization. J Mater Sci Technol 66:82–90. doi:10.1016/j.jmst.2020.03.089

[53] Wang P, Yu Z, Li B, Cai X, Zeng Z, Chen X, Wang X. 2015. Development of an efficient conjugation-based genetic manipulation system for Pseudoalteromonas. Microb Cell Fact 14:11. doi:10.1186/s12934-015-0194-8 25612661 PMC4318363

[54] Zeng ZS, Guo XP, Cai XS, Wang PX, Li BY, Yang JL, Wang XX. 2017. Pyomelanin from Pseudoalteromonas lipolytica reduces biofouling. Microb Biotechnol 10:1718–1731. doi:10.1111/1751-7915.12773 28834245 PMC5658579

[55] Sajadi E, Fatemi S-A, Babaeipour V, Deldar AA, Yakhchali B, Anvar MS. 2019. Increased cellulose production by heterologous expression of bcsA and B genes from Gluconacetobacterxylinus in E. coli Nissle 1917. Bioprocess Biosyst Eng 42:2023–2034. doi:10.1007/s00449-019-02197-4 31489493

[56] Sperandio V, Torres AG, Kaper JB. 2002. Quorum sensing Escherichia coli regulators B and C (QseBC): a novel two-component regulatory system involved in the regulation of flagella and motility by quorum sensing in E. coli. Mol Microbiol 43:809–821. doi:10.1046/j.1365-2958.2002.02803.x 11929534

[57] Zeng Z, Gu J, Lin S, Li Q, Wang W, Guo Y. 2023. Molecular basis of the phenotypic variants arising from a Pseudoalteromonas lipolytica mutator. Microb Genom 9:001118. doi:10.1099/mgen.0.001118 37850970 PMC10634453

[58] Wang WQ, Tang KH, Wang PX, Zeng ZS, Xu T, Zhan W, Liu TL, Wang Y, Wang XX. 2022. The coral pathogen Vibrio coralliilyticus kills non-pathogenic holobiont competitors by triggering prophage induction. Nat Ecol Evol 6:1132–1144. doi:10.1038/s41559-022-01795-y 35773344

