# Supplementary material for: A point mutation in a wspF-like gene in Pseudoalteromonas lipolytica enhances the anticorrosion activity
Source: Appl Environ Microbiol. 2025 Jan 28;91(2):e02154-24. doi: 10.1128/aem.02154-24 (PMC11837571; doi:10.1128/aem.02154-24)

**SUPPLEMENTARY FILE**

**A point mutation in *wspF-like* gene in *Pseudoalteromonas lipolytica* enhances the anticorrosion activity**

Zhenshun Zeng^1^*, Dan He^1^, Zhiying Zhao^2^, Tianci He^1^, Qian Li^1^, Yuqi Wang^1^*

^1^Key Laboratory for Water Quality and Conservation of the Pearl River Delta, Ministry of Education; School of Environmental Science and Engineering, Guangzhou University, Guangzhou 510006, China

^2^State Key Laboratory of Applied Microbiology Southern China, Guangdong Provincial Key Laboratory of Microbial Culture Collection and Application, Guangdong Institute of Microbiology, Guangdong Academy of Sciences, Guangzhou 510070, China

*To whom correspondence should be addressed. E-mail: [zzshun@gzhu.edu.cn](mailto:zzshun@gzhu.edu.cn); [yqwang@gzhu.edu.cn](mailto:yqwang@gzhu.edu.cn);

Tel. +86 20 89366943.

**Table S1. Sequences of primers used in this study.**

| **Primer name** | **Sequence** |
| --- | --- |
| **In-frame deletions** |  |
| V08765-F | agtcactggggatcctctagaATTGGTCAAGAAGAGGTGGTGATAA |
| V08765-R | gccagtgccaagcttgcatgcATGACCGCACCTAAAGTTCCG |
| **Complementation** |  |
| p-08765-F | CGGAATTCGATGGTTATTGTGGGTGAG |
| p-08765-R | ACGCGTCGACTTATCCACAGCCAGTCTCA |
| p-08765-motA-F | CGGAATTCGATGGTTATTGTGGGTGAG |
| p-08765-motA-R | ACGCGTCGACTGCTGGCTTTTTTGAGAGG |
| **Sequencing primers** |  |
| V08765-seqF | TTTACTGCAAGGTACACCA |
| V08765-seqR | TGAACAAAGCACTGACAAC |
| pBBR1MCS-f | TCGTTAAATAGCCGCTTATG |
| pBBR1MCS-r | AATTTCACACAGGAAACAGC |

**Table S2. Chemical composition of Q235 carbon steel (wt%).**

| Elements | C | Si | Mn | P | S | Al | Fe |
| --- | --- | --- | --- | --- | --- | --- | --- |
| Content (wt%) | 0.2 | 0.2 | 0.2 | 0.03 | 0.03 | 0.03 | Balance |

**Figure S1.** Attached products on the glass surface were examined by SEM at two different magnitudes after static incubation for 7 days. Elemental mapping showed that the aggregates of the wild-type and Δ*08765(707A)* strains comprised approximately 24.72% and 34.02% calcium (wt%).


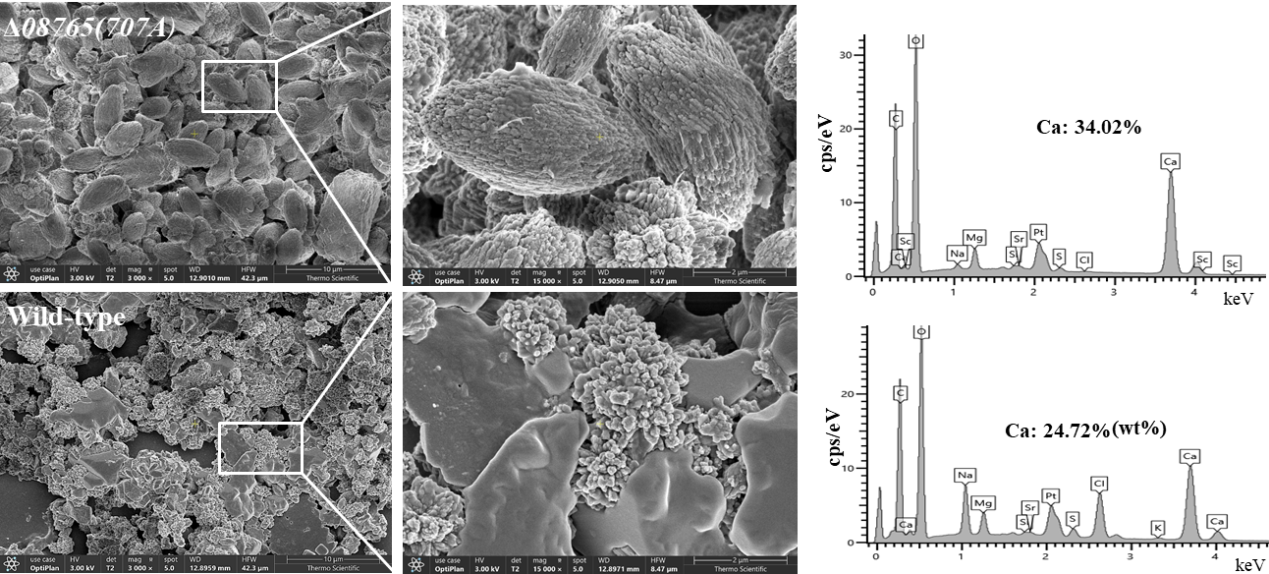


**Figure S2.** Biofilms stained with the LIVE/DEAD viability stain were examined on glass surface by CLSM after static incubation with Δ*08765* strain for1 day.


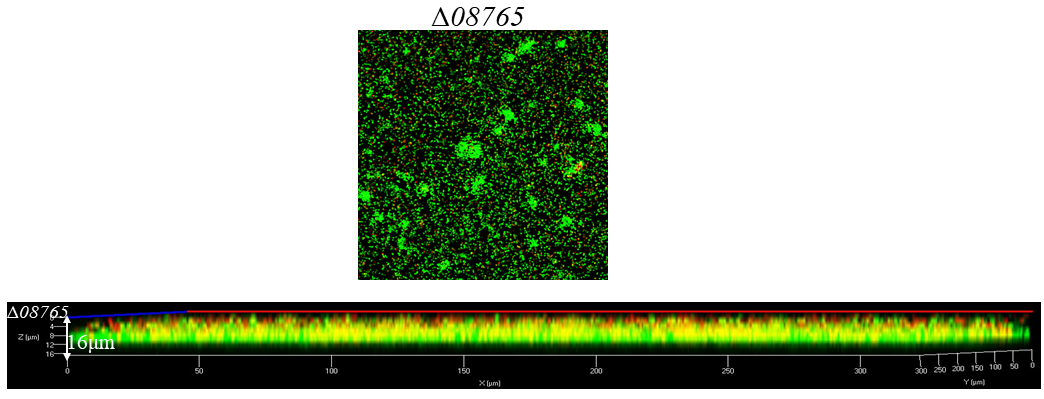


**Figure S3.** (**A**) Colony morphologies were shown for Δ*flhA* and Δ*fleQ* on SWLB agar medium. (**B**) Swimming motility was examined for Δ*flhA* and Δ*fleQ*. (**C**) Pitting corrosion on the steel surface was examined by optical profilometry after performing an immersion test.


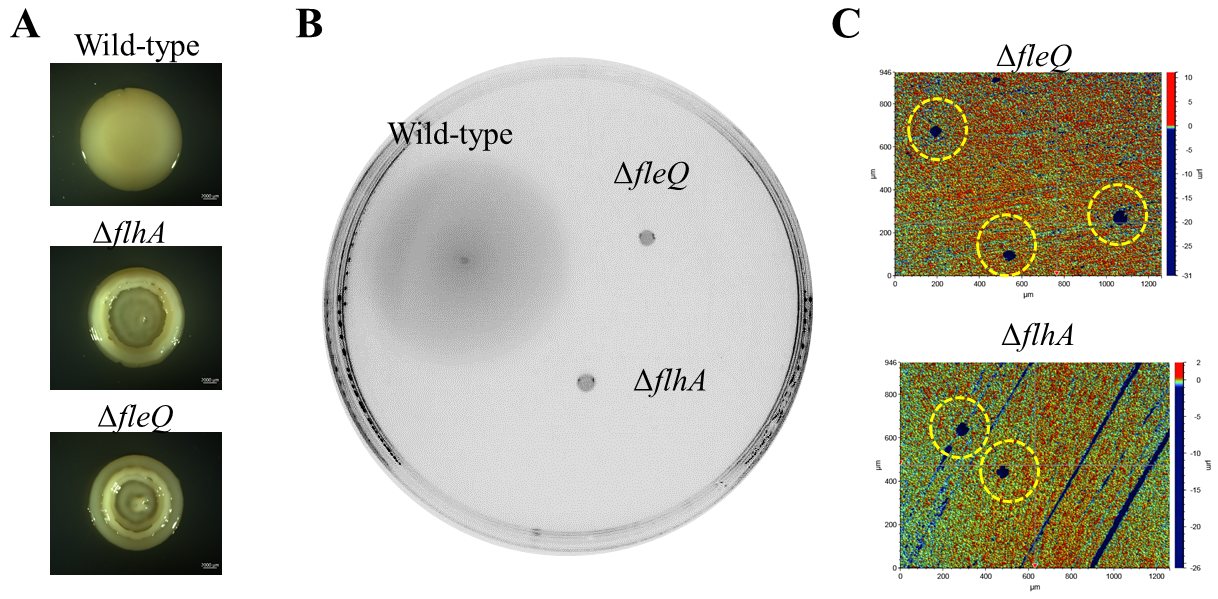

Supplement: Supplemental material — Tables S1 and S2; Figures S1 to S3. [file aem.02154-24-s0001.docx]
